# Utilization Patterns and Outcomes of People With Diabetes and COVID-19: Evidence From United States Medicare Beneficiaries in 2020

**DOI:** 10.3389/fcdhc.2022.920478

**Published:** 2022-07-05

**Authors:** Andrea M. Austin, Christopher G. Leggett, Peter Schmidt, Paul Bolin, Eugene C. Nelson, Brant J. Oliver, Ashleigh C. King

**Affiliations:** ^1^ The Dartmouth Institute for Health Policy and Clinical Practice, Geisel School of Medicine at Dartmouth, Hanover, NH, United States; ^2^ Grossman School of Medicine, New York University, New York, NY, United States; ^3^ Brody School of Medicine, East Carolina University, Greenville, NC, United States; ^4^ Departments of Community & Family Medicine and Psychiatry, Geisel School of Medicine at Dartmouth, Hanover, NH, United States; ^5^ Office of Patient Experience, Value Institute, Dartmouth-Hitchcock, Lebanon, NH, United States

**Keywords:** medicare, diabetes, COVID-19, utilization, public health

## Abstract

**Objective:**

Determine differences in utilization patterns, disease severity, and outcomes between patients with and without diabetes mellitus diagnosed with COVID-19 in 2020

**Research Design and Methods:**

We used an observational cohort comprised of Medicare fee-for-service beneficiaries with a medical claim indicating a COVID-19 diagnosis. We performed inverse probability weighting between beneficiaries with and without diabetes to account for differences in socio-demographic characteristics and comorbidities.

**Results:**

In the unweighted comparison of beneficiaries, all characteristics were significantly different (P<0.001). Beneficiaries with diabetes were younger, more likely to be black, had more comorbidities, higher rates of Medicare-Medicaid dual-eligibility, and were less likely to be female. In the weighted sample, hospitalization rates for COVID-19 among beneficiaries with diabetes was higher (20.5% vs 17.1%; p < 0.001). Outcomes of hospitalizations were similarly worse among beneficiaries with diabetes: admissions to ICU during hospitalizations (7.78% vs. 6.11%; p < 0.001); in-hospital mortality (3.85% vs 2.93%; p < 0.001); and ICU mortality (2.41% vs 1.77%). Beneficiaries with diabetes had more ambulatory care visits (8.9 vs. 7.8, p < 0.001) and higher overall mortality (17.3% vs. 14.9%, p < 0.001) following COVID-19 diagnosis.

**Conclusion:**

Beneficiaries with diabetes and COVID-19 had higher rates of hospitalization, ICU use and overall mortality. While the mechanism of how diabetes impacts the severity of COVID-19 may not be fully understood, there are important clinical implications for persons with diabetes. A diagnosis of COVID-19 leads to greater financial and clinical burden than for their counterparts, persons without diabetes, including perhaps most significantly, higher death rates.

## Introduction

The current COVID-19 pandemic, caused by infection and sequelae of the SARS-CoV-2 virus, continues to pose human health risks and threats. Surveillance of the demographic characteristics and outcomes of the Medicare population diagnosed with COVID-19 is important for understanding the epidemiology of the disease given that 78% of COVID-19 mortality, or 559,309 of 721,465 deaths reported through 14 October 2021, in the US have occurred in individuals over 65 (1). As of May 21, 2021 the Centers of Medicare and Medicaid Services reported that rural areas, females, and Medicare-Medicaid dual-eligible beneficiaries are disproportionately impacted by COVID-19 (1).

A cohort study in Louisiana found that a disproportionate number of patients dying in the hospital from COVID-19 were black, but that race did not appear to be as significant a predictor of mortality from COVID-19 as originally reported (2). Similar results were found in a nationwide study comparing black Medicare beneficiaries to white beneficiaries (3). Dementia, disproportionately prevalent in Black, Hispanic, and female Medicare beneficiaries, has been linked with the highest rates of infection and mortality among Medicare beneficiaries (4).

Perhaps the most prevalent comorbid condition that impacts the pathway to recovery for patients diagnosed with COVID-19 is diabetes mellitus (5–7). Diabetes affects 10.5% of the US population and in 2019 was responsible for 87,647 deaths (2). Furthermore, several meta-analyses have found the prevalence rate of diabetes in patients with COVID-19 to be slightly higher, ranging from 11.5%-31% (8–10)., which may be due to an increased susceptibility to COVID-19 among diabetics (11). Diabetes and hypertension (a common predictor and exacerbator of diabetes) were the two most common comorbidities found in the study of the impact of COVID-19 on demented patients (4). Clinical data from across the world suggests people with diabetes experience 2-3 times more severe clinical outcomes including hospitalizations and death with a COVID-19 diagnosis (5, 10). Understanding the impact of diabetes on older COVID-19 patients living in the United States (U.S.) is therefore important to develop future pathways of prevention and treatment in this vulnerable population.

We report an analysis of a nationwide cohort of U.S. Medicare beneficiaries with and without diabetes diagnosed with COVID-19 during 2020. We analyze the impact on disease trajectories (utilization and outcomes) in beneficiaries with and without diabetes, before and after adjusting for socio-demographic characteristics and comorbidities. The COVID-19 vaccine became available in December 2020 (12). Therefore, looking at all the diagnosed population and their utilization patterns and outcomes in 2020 will provide a comprehensive picture of the disease trajectory among Medicare beneficiaries throughout the country prior to widespread vaccination in Medicare populations with and without diabetes.

## Research Design and Methods

### Data Source, Study Population, and Covariates

We conducted an observational cohort study using a complete national sample of beneficiaries with 12 months of enrollment in fee-for-service (FFS) Medicare during 2020. If a beneficiary turned 65 during 2020, they needed to have consistent FFS Medicare enrollment from the month of their date of birth through the end of the year. Conversely, any beneficiary who died during 2020 need to have consistent enrollment from January through their month of death to be included in the FFS cohort. We included all FFS patients who were newly diagnosed with COVID-19andwere U.S. residents 65 or older at the time of diagnosis. We did not include Medicare Advantage patients. We excluded patients younger than 65 since their basis for Medicare eligibility is due to disability or End-stage Renal Disease making these populations significantly different from the aged Medicare population.

We gathered baseline health characteristics from the 2020 Medicare claims data, including patient-level comorbidities identified using the Elixhauser Comorbidity Software developed by the Agency for Healthcare Research and Quality (AHRQ) as part of the Healthcare Cost and Utilization Project (HCUP). Our main exposure variable of interest, diabetes mellitus, was identified using the Elixhauser algorithm (13, 14). We used the Medicare Master Beneficiary Summary File (MBSF) to determine each patient’s sex, race, age at diagnosis, Medicare-Medicaid dual-eligibility status, and ZIP code. The ZIP code was then used to establish rurality (urban vs. rural), Census region, and median household income (15).

### Outcome Measures

We identified outcomes for the cohort for the remainder of 2020 following their index COVID-19 diagnosis. Outcomes studied included total number of hospitalizations to an acute care or critical access hospital with a primary diagnosis of COVID-19, number of intensive care unit (ICU) admissions, number of ambulatory care visits, average spending on COVID-19 related hospitalizations, prescriptions for drugs commonly used to treat COVID-19, and death (in hospital, in the ICU, and overall). Death dates were obtained from the Medicare MBSF while ambulatory care visits were determined using Current Procedure Terminology codes 99202-99499 in the Part B and Outpatient files. Hospitalizations, ICU visits, and ICU deaths were determined using the Inpatient Claim and Revenue Center files, with revenue center codes 0200-0209 used to determine ICU use. To calculate average spending, we used the methods in the codebook for the Medicare Inpatient file, which involves multiplying the daily per diem amount by the number of Medicare-covered days and then adding result to the claim payment amount (16). The list of drugs used for treatment of COVID-19 included prescription fills from the Medicare Part D data for dexamethasone, hydroxychloroquine, interferon, ivermectin, lopinavir/ritonavir, and tocilizumab.

### Statistical Analysis

We applied traditional statistical methods to describe the demographic and health characteristics of the cohort, including means and standard deviations for continuous variables (spending on COVID-19 related hospitalizations, median household income, and comorbidity count), and counts and percentages for categorical variables (age categories, sex, race, diabetes status, Medicare-Medicaid dual-eligibility status, urban/rural indicator, and all outcomes).

We then calculated an inverse probability weights (IPW) to control for confounding. The process of creating IPWs involved applying a logistic regression, with the exposure, diabetes status, regressed on the following covariates: 1. baseline demographic characteristics including the patient’s age at diagnosis; 2. sex; 3. race; 4. Medicare-Medicaid dual-eligibility status; 5. rural or urban residence; 6. census region; and 7. individual disease flags given by the Elixhauser Comorbidity definitions (excluding diabetes). The inverse of the predicted probability of diabetes from this regression was used to calculate an IPWforeach patient. To confirm that the IPWs created covariate balance, the weights were used in comparing the exposed and unexposed group with respect to baseline demographics and comorbidities, with standardized mean differences (SMD) used to assess balance. These IPWs were applied when comparing outcomes across the two groups (patients with and without diabetes) to control for confounding (17–19).

All analyses were performed using SAS (version 7.15), with the logistic regression model, used to create IPWs, run using the LOGISTIC procedure. Unless otherwise indicated, a threshold of p < 0.001 was used for significance.

## Results

Among the 1,439,520 Medicare beneficiaries with a COVID-19 diagnosis present in the 2020 Medicare claims, 469,448 (33%) had concurrent diabetes mellitus (**Table 1**). In the unweighted comparison of patients with and without diabetes, all patient characteristics were significantly different. In particular, patients with diabetes were younger, were more likely to be black, had a higher number of comorbid diseases, had higher rates of Medicare-Medicaid dual-eligibility, and were less likely to be female than patients without diabetes. After propensity weighting, all baseline covariates were balanced across diabetes status and no longer statistically significant with standardized values within the -0.1 to 0.1 range (**Table 2**) (17–19). In the weighted cohort, 56.5% of patients were female, 90.4% of patients were non-black, 39% of patients were from the South, and 24% from the Midwest.

**Table 1:** Unweighted demographic characteristics of Medicare beneficiaries with a diagnosis of Covid-19 in the 2020 claims data.

	All		Diabetes		No Diabetes		P-value
	N/Mean	%/SD	N/Mean	%/SD	N/Mean	%/SD	
All	1,439,520		469,448		970,072		
Female	822,633	57.1%	247,269	52.7%	575,364	59.3%	<0.0001
Age							
65-74	635,433	44.1%	204,554	43.60%	430,879	44.40%	<0.0001
75-84	464,465	32.3%	168,912	36.00%	295,553	30.50%	<0.0001
85+	339,622	23.6%	95,982	20.40%	243,640	25.10%	<0.0001
Non-Black	1,307,184	90.8%	403,390	85.90%	903,794	93.20%	<0.0001
Medicare-Medicaid Dual-Eligible	404,131	28.1%	175,266	37.30%	228,865	23.60%	<0.0001
Median Household Income	$49647.80	$20607.80	$48715.8	$19833.9	$50098.9	$20957.1	<0.0001
Urban	1,108,866	77.0%	363,814	77.5%	745,052	76.8%	<0.0001
Elixhauser total	3.75	3.29	6.03	3.35	2.65	2.62	<0.0001
Region							
Northeast	296,928	20.6%	96,276	20.50%	200,652	20.70%	0.0145
South	565,872	39.3%	192,570	41.0%	373,302	38.5%	<0.0001
West	228,428	15.9%	72,374	15.40%	156,054	16.10%	<0.0001
Midwest	348,292	24.2%	108,228	23.10%	240,064	24.70%	<0.0001

**Table 2:** Weighted demographic characteristics of Medicare beneficiaries with a diagnosis of COVID-19 in the 2020 claims data.

	Diabetes		No Diabetes		Standardized Differences
	N/Mean	%/SD	N/Mean	%/SD	
Female	795,077	56.50%	824,162	56.90%	-0.0080
Age					
65-74	583,240	41.40%	632,077	43.60%	-0.0443
75-84	461,054	32.70%	471,455	32.50%	0.0046
85+	363,569	25.80%	345,687	23.90%	0.0456
Non-Black	1,272,661	90.40%	1,310,416	90.40%	-0.0009
Medicare-Medicaid Dual-Eligible	418,004	29.70%	415,200	28.60%	0.0229
Median Household Income	$49,612.10	$34,805.80	$49,611.50	$25,417.90	0.0000
Urban	1,075,491	76.40%	1,121,668	77.40%	-0.0239
Elixhauser total	4.83	5.25	3.33	3.72	0.3310
Region					
Northeast	295,878	21.00%	299,958	20.70%	0.0078
South	552,501	39.20%	569,939	39.30%	-0.0017
West	218,774	15.50%	228,273	15.80%	-0.0058
Midwest	340,710	24.20%	351,049	24.20%	-0.0005

After propensity weighting, we observed meaningful but not consistently statistically significant differences in all utilization measures. Beneficiaries with diabetes had higher rates of each outcome than beneficiaries without diabetes except in the use of drug treatments for COVID-19 (**Table 3**). In the weighted sample, the rate of hospitalization for COVID-19 among beneficiaries with diabetes (20.5%) and beneficiaries without diabetes (17.1%) were significant but the effect size was limited, with a standardized mean difference (SMD) of 0.09 (p<0.0001). Similarly, admission to ICU during hospitalizations was also significantly higher among beneficiaries with diabetes vs. beneficiaries without diabetes (7.78% vs. 6.11%), again with a limited effect size (SMD = 0.07, p<0.0001). Beneficiaries with diabetes had an average of 1.1 more ambulatory care encounters following their COVID-19 diagnosis (8.9 vs. 7.8; SMD = 0.04, p<0.0001). Beyond higher utilization, beneficiaries with diabetes had higher rates of overall mortality in 2020 following their COVID-19 diagnosis than beneficiaries without diabetes (17.3% vs. 14.9%; SMD = 0.04, p<0.0001).

**Table 3:** Weighted measures of Medicare utilization among beneficiaries with a prior diagnosis of COVID-19 in the 2020 claims data.

	Diabetes		No Diabetes		Standardized Differences	P-Value*
	N/Mean	%/SD	N/Mean	%/SD		
Total Hospitalizations for COVID	289,076	20.50%	248,457	17.10%	0.0867	<0.0001
ICU Admissions	109,500	7.78%	88,575	6.11%	0.0656	<0.0001
Death in Hospital	54,272	3.85%	42,451	2.93%	0.0512	<0.0001
Death in ICU	33,991	2.41%	25,692	1.77%	0.0448	<0.0001
Any Death	243,436	17.30%	215,796	14.90%	0.0654	<0.0001
Average # Ambulatory Care Visits per beneficiary Post-COVID Diagnosis	8.9	30.91	7.84	18.84	0.0412	<0.0001
Spending on COVID-related Hospitalization	$20,501	$31,290	$19,413	$24,201	0.0389	<0.0001
Drug Treatments for COVID						
Dexamethasone	95,056	6.75%	91,635	6.32%	0.0173	<0.0001
Hydroxychloroquine	18,770	1.33%	20,350	1.40%	-0.0061	0.7243
Interferon	116	0.01%	196	0.01%	-0.0051	0.5000
Ivermectin	6,107	0.43%	6,081	0.42%	0.0022	0.4662
Lopinavir/ritonavir	227	0.02%	256	0.02%	-0.0012	0.5000
Tocilizumab	83	0.01%	83	0.01%	0.0002	0.5000

*P-values have been calculated without using inverse propensity weights.

Only steroid dexamethasone used for treatment of COVID-19 showed a significant difference between patients with and without diabetes (6.75% vs 6.32%: SMD = 0.02, p<0.0001). Other drugs evaluated were not significantly different in their application (**Table 3**). Differences in usage were related to regional rates of prescribing more than a disease comorbidity indicator. Whether a patient was prescribed these treatments for COVID-19 was impacted more by where they reside than an underlying risk factor for disease severity such as diabetes.

## Conclusions

In this study, we compared treatment patterns and outcomes for patients with and without diabetes following the time of their diagnosis of COVID-19 in 2020. We found that patients with diabetes had more comorbid conditions than those without, were more likely to be male, and were more likely to be Medicare-Medicaid dual-eligible. When controlling for these differences through the application of inverse probability weighting, we saw that patients with diabetes still had a greater likelihood of hospitalization for COVID-19, that their hospitalizations would be more costly overall, and they had a greater overall likelihood of death following their COVID-19 diagnosis. These findings indicate that patients with diabetes had worse trajectories in terms of physical and financial burden in the treatment of COVID-19 compared to socio-demographically similar patients without diabetes. Additionally, studies have shown that access to preventative and primary care is important in the management of diabetes (20). In the context of the COVID-19 pandemic, preventative care was often canceled, which likely impacted people with diabetes and their ability to effectively control their disease.

Other studies of patients with diabetes and COVID-19 have shown differences in mortality and severity of illness for patients with diabetes compared to those without, with one meta-analysis describing a two-fold increase in mortality (21). Some studies are skeptical about the relationship between diabetes and COVID-19 trajectories and call for more concrete evidence (22, 23). A strength of the study is that we address some gaps in evidence for elderly patients with diabetes in the United States using a nationwide sample of FFS Medicare beneficiares. Our study has several notable limitations. The first is with respect to the drugs used to treat COVID-19; there are other medically indicated reasons for using many of these drugs (24–26). While we only looked at usage following COVID-19 diagnosis, due to the nature of using Medicare claims, we cannot be certain that the drugs were prescribed specifically to treat COVID-19. A second limitation is our inability to use the most current data, to analyze results for 2021. Additional burdens to COVID-19 patients include post-acute COVID-19 syndrome, or the long-term repercussions of infection, including impacts to physical and mental health such as fatigue, declines in quality of life, and chronic kidney disease (27). Another limitation is that our study included only people who qualified for Medicare on the basis of old age (65+) so is not generalizable to younger populations of people with diabetes. Finally, the roll-out of vaccines in late 2020 and into 2021 for COVID-19 – with many states prioritizing medically complex patients, including patients with diabetes – will be important to consider in future work. For example, it will be important to determine if it improved the trajectory of patients with diabetes, in particular, those with breakthrough COVID-19 infections following vaccination.

In conclusion, we have shown that Medicare beneficiaries with diabetes have higher rates of hospitalization and ICU use than similar beneficiaries without diabetes. They also have higher rates of mortality overall, and higher rates of dying once hospitalized for COVID-19. While the mechanism of how diabetes impacts the severity of COVID-19 in patients (leading to higher utilization and mortality) is not yet fully understood, there are many clinical implications.

## Data Availability Statement

The data analyzed in this study is subject to the following licenses/restrictions: An active Data Use Agreement with CMS is required to access the 2020 Medicare data used in this analysis.

## Ethics Statement

The studies involving human participants were reviewed and approved by Committee for the Protection of Human Subjects - Dartmouth College. Written informed consent for participation was not required for this study in accordance with the national legislation and the institutional requirements.

## Author Contributions

AA and AK wrote the manuscript. AK and CL had full access to all the data and performed data and statistical analyses with statistical expertise provided by AA, CL, PS, PB, EN and BO edited and reviewed the manuscript and contributed to discussion. EN is the guarantor of this work. All authors contributed to the article and approved the submitted version.

## Funding

This work was supported by the State of North Carolina under Session Law 2020-4, contract 98-00.

## Conflict of Interest

The authors declare that the research was conducted in the absence of any commercial or financial relationships that could be construed as a potential conflict of interest.

## Publisher’s Note

All claims expressed in this article are solely those of the authors and do not necessarily represent those of their affiliated organizations, or those of the publisher, the editors and the reviewers. Any product that may be evaluated in this article, or claim that may be made by its manufacturer, is not guaranteed or endorsed by the publisher.

## References

[1] Preliminary Medicare COVID-19 Data Snapshot (2021). Available at: https://www.cms.gov/files/document/medicare-covid-19-data-snapshot-fact-sheet.pdf.

[2] Price-Haywood EG Burton J Fort D Seoane L. Hospitalization and Mortality Among Black Patients and White Patients With Covid-19. N Engl J Med (2020) 382(26):2534–43. doi: 10.1056/NEJMsa2011686 PMC7269015 32459916

[3] Asch DA Islam MN Sheils NE Chen Y Doshi JA Buresh J. Patient and Hospital Factors Associated With Differences in Mortality Rates Among Black and White US Medicare Beneficiaries Hospitalized With COVID-19 Infection. JAMA Network Open (2021) 4(6):e2112842. doi: 10.1001/jamanetworkopen.2021.12842 34137829 PMC11849740

[4] Lamont H Samson LW Zuckerman R Dey J Oliveira I Tarazi W. The Impact of COVID-19 on Medicare Beneficiaries With Dementia (Issue Brief). Washington, DC: Office of the Assistant Secretary for Planning and Evaluation, US Department of Health and Human Services (2021).

[5] Sourij H Aziz F Bräuer A Ciardi C Clodi M Fasching P. COVID-19 Fatality Prediction in People With Diabetes and Prediabetes Using a Simple Score Upon Hospital Admission. Diabetes Obes Metab (2021) 23(2):589–98. doi: 10.1111/dom.14256 PMC7753560 33200501

[6] Reuters. Out Of Control: America's Losing Battle Against Diabetes (2021). Available at: https://www.reuters.com/investigates/special-report/usa-diabetes-covid/ [Accessed October 14, 2021].

[7] Riddle MC Buse JB Franks PW Knowler WC Ratner RE Selvin E. COVID-19 in People With Diabetes: Urgently Needed Lessons From Early Reports. Diabetes Care (2020) 43(7):1378–81. doi: 10.2337/dci20-0024 PMC7305002 32409505

[8] Singh AK Gillies CL Singh R Singh A Chudasama Y Coles B. Prevalence of Co-Morbidities and Their Association With Mortality in Patients With COVID-19: A Systematic Review and Meta-Analysis. Diabetes Obes Metab (2020) 22(10):1915–24. doi: 10.1111/dom.14124 PMC7361304 32573903

[9] Barrera FJ Shekhar S Wurth R Moreno-Pena PJ Ponce OJ Hajdenberg M. Prevalence of Diabetes and Hypertension and Their Associated Risk for Poor Outcomes in COVID-19 Patients. J Endo Soc (2020) 4(9):1–16. doi: 10.1210/jendso/bvaa102 PMC7454711 32885126

[10] Bradley SA Banach M Alvarado N Smokovski I Bhaskar SMM. Prevalence and Impact of Diabetes in Hospitalized COVID-19 Patients: A Systematic Review and Meta-Analysis. J Diabetes (2021) 14(2):144–57. doi: 10.1111/1753-0407.13243 PMC9060142 34939735

[11] Goyal A Gupta Y Kalaivani M Praveen PA Ambekar S Tandon N. SARS-Cov-2 Seroprevalence Inindividuals With Type 1 and Type 2 Diabetes Compared With Controls. Endocr Pract (2022) 28(2):191–8. doi: 10.1016/j.eprac.2021.12.009 PMC8669945 34920109

[12] Wall Street Journal. First Covid-19 Vaccine Given to U.S. Public (2020). Available at: https://www.wsj.com/articles/covid-19-vaccinations-in-the-u-s-slated-to-begin-monday-11607941806 [Accessed October 14, 2021].

[13] Quan H Sundararajan V Halfon P Fong A Burnand B Luthi J. Coding Algorithms for Defining Comorbidities in ICD-9-CM and ICD-10 Administrative Data. Med Care (2005) 43(11):1130–9. doi: 10.1097/01.mlr.0000182534.19832.83 16224307

[14] Elixhauser Comorbidity Software Refined for ICD-10-CM Healthcare Cost and Utilization Project (HCUP). In: Agency for Healthcare Research and Quality, Rockville, Md. Available at: www.hcup-us.ahrq.gov/toolssoftware/comorbidityicd10/comorbidity_icd10.jsp. 21413206

[15] Agency for Healthcare Research and Quality. Elixhauser Comorbidity Software Refined for ICD-10-CM Healthcare Cost and Utilization Project (HCUP) (2021). https://www.hcup-us.ahrq.gov/toolssoftware/comorbidityicd10/ElixhauserComorbidity_v2021-1.zip [Accessed August 11, 2021].

[16] Claim (Medicare) Payment Amoutn (2022). Available at: https://resdac.org/cms-data/variables/claim-medicare-payment-amount.

[17] Austin PC. Assessing Covariate Balance When Using the Generalized Propensity Score With Quantitative or Continuous Exposures. Stat Methods Med Res (2019) 28(5):1365–77. doi: 10.1177/0962280218756159 PMC6484705 29415624

[18] Austin PC. An Introduction to Propensity Score Methods for Reducing the Effects of Confounding in Observational Studies. Multivar Behav Res (2011) 46(3):399–424. doi: 10.1080/00273171.2011.568786 PMC3144483 21818162

[19] Austin PC Stuart EA. Moving Towards Best Practice When Using Inverse Probability of Treatment Weighting (IPTW) Using the Propensity Score to Estimate Causal Treatment Effects in Observational Studies. Stat Med (2015) 34(28):3661–79. doi: 10.1002/sim.6607 PMC4626409 26238958

[20] Virnig BA Shippee ND O'Donnell B Zeglin J Parashuram S. Use of and Access to Health Care by Medicare Beneficiaries With Diabetes: Impact of Diabetes Type and Insulin Use, 2007-2011. In: Data Points Publication Series 2014; Data Points #18. Rockville, MD: Agency for Healthcare Research and Quality (2014). 24872962

[21] Kumar A Arora A Sharma P Anikhindi SA Bansal N Singla V. Is Diabetes Mellitus Associated With Mortality and Severity of COVID-19? A Meta-Analysis. Diabetes Metab syndrome (2020) 14(4):535–45. doi: 10.1016/j.dsx.2020.04.044 PMC7200339 32408118

[22] Tadic M Cuspidi C Sala C. COVID-19 and Diabetes: Is There Enough Evidence? J Clin Hypertens (Greenwich Conn) (2020) 22(6):943–8. doi: 10.1111/jch.13912 PMC7300807 32472662

[23] Hussain A Bhowmik B do Vale Moreira NC. COVID-19 and Diabetes: Knowledge in Progress. Diabetes Res Clin Pract (2020) 162:108142. doi: 10.1016/j.diabres.2020.108142 32278764 PMC7144611

[24] Johnson DB Lopez MJ Kelley B. Dexamethasone. In: StatPearls. Treasure Island (FL: StatPearls Publishing Copyright © 2021, StatPearls Publishing LLC (2021).

[25] Singh TU Parida S Lingaraju MC Kesavan M Kumar D Singh RK. Drug Repurposing Approach to Fight COVID-19. Pharmacol. Rep. PR (2020) 72(6):1479–508. doi: 10.1007/s43440-020-00155-6 PMC7474498 32889701

[26] Fox LM. Ivermectin: Uses and Impact 20 Years on. Curr. Opin. Infect. Dis (2006) 19(6):588–93. doi: 10.1097/QCO.0b013e328010774c 17075336

[27] Nalbandian A Sehgal K Gupta A Madhavan MV McGroder C Stevens JS. Post-Acute COVID-19 Syndrome. Nat. Med (2021) 27(4):601–15. doi: 10.1038/s41591-021-01283-z PMC8893149 33753937

